# Bibliographical

**Published:** 1897-04

**Authors:** 


### Bibliographical.

A Practical Treatise on Artificial Crown and Bridge-Work, by George Evans. Lecturer on Crown and Bridge-Work in the Baltimore College of Dental Surgery; Member of the American Dental Association, Member of the Southern Dental Association, Member of the Dental Society of the State of New York, etc., etc. Fifth Edition, Revised; 625 Illustrations.

That a fifth edition of this work should be so soon called for fully attests its value and popularity, and also the rapid progress that is being made in the special subjects upon which it treats—crown and bridge-work. No man has done more to develop this branch of dental practice than Dr. Evans. It has received a large share of his professional attention for many years, indeed, he has given more and closer attention to this subject than any other man in this country. He is constantly devising new points and methods, and improving those that have been in use, thus making this variety of work more practicable and giving it a wider range of application and usefulness.

The results of all this effort is embodied in this volume as it was in the former editions at the time of their respective issues. Dr. Evans very generously and properly gives credit to all who have, in any way, contributed to the success of his work; he has, in many instances, presented methods and processes devised by others that, but for him, would not have been known to the profession at all.

The methods given in this work would seem to meet every conceivable case, and in a very efficient manner. It is profusely illustrated, and in such a manner that every illustration conveys some important point or lesson.

No one who attempts crown or bridge-work can afford to be without this book. It is published by the S. S. White Manufacturing Company, and this is enough to say about the mechanical execution of the work. It can be obtained of the dental supply houses, or of the booksellers, as well as of the publishers.

---

A Compend of Dental Pathology and Therapeutics, by Henry H. Burchard, M.D., D.D.S. Special Lecturer upon Dental Pathology and Therapeutics in the Philadelphia Dental College.

This is a little work of about one hundred and forty pages, presenting the two subjects given in the caption, Pathology and Therapeutics, in the form of questions and answers. For this method of studying these subjects this work seems to be well prepared, and will be valuable especially to beginners, and will prepare them for a more extended study of these branches.

There is a need for elementary text-books in our colleges and this form is probably as practicable as can be devised. The objection has been made to quiz compends generally that students are too much inclined to rest satisfied with that which they have gained by this means, whereas, it should, in no case, be more than an introduction to the subject in hand. This work comes as near meeting this objection, if not more so, than any work of the kind that has come to notice. It will be helpful to every student who will use it as it is intended to be used.

It is a work that should be in the hands of every student, and it will he helpful to many a busy practitioner as well.

It is published by the S. S. White Dental Manufacturing Company, and is well gotten up in every particular.

